# Supplementary material for: Identification of photoperiod-induced specific miRNAs in the adrenal glands of Sunite sheep (Ovis aries)
Source: Front Vet Sci. 2022 Jul 22;9:888207. doi: 10.3389/fvets.2022.888207 (PMC9354845; doi:10.3389/fvets.2022.888207)
Supplement: Supplementary file 1 [file Data_Sheet_1.ZIP › Supplementary file/miRDeep2 file.zip!/miRDeep2 file/Know_Structure/SPLP42A2/oar-mir-29b-1.pdf]

miRBase precursor : oar-mir-29b-1
Total read count : 12485
oar-miR-29b read count : 11694
remaining reads : 791

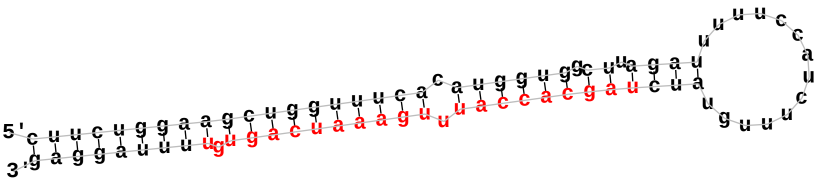

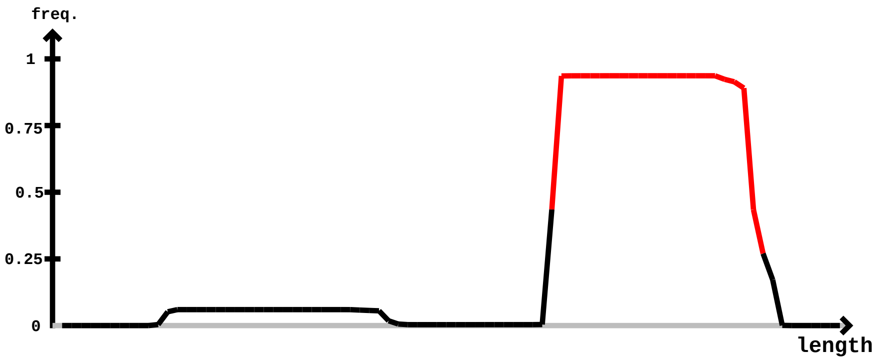

oar-miR-29b

5'- cuucuggaagcugguuucacaugguggcuuagauuuuuccaucuuuguaucuagcaccauuugaaaucaguguuuuaggag -3'	exp		
(((((((((((((((((((.((((((.((.((((..............)))))))))))).)))))))))).)))))))))	reads	mm	sample
.........gcugguuucacaugguggcu..	1	0	seq
.........gcugguuucacaugguggcuu...	6	0	seq
.........gcugguuucacaugguggcuua..	7	0	seq
.........gcugguuucacaugguggcuuag...	3	0	seq
.........gcugguuucacaugguggcuuaga..	31	0	seq
.........gcugguuucacaugguggcuuagauu..	1	0	seq
..........cugguuucacaugguggc...	1	0	seq
..........cugguuucacaugguggcuu...	13	0	seq
..........cugguuucacaugguggcuua..	11	0	seq
..........cugguuucacaugguggcuuag...	9	0	seq
..........cugguuucacaugguggcuuaga..	424	0	seq
..........cugguuucacaugguggcuuagau...	120	0	seq
..........cugguuucacaugguggcuuagauu..	18	0	seq
...........ugguuucacaugguggcuu...	1	0	seq
...........ugguuucacaugguggcuuag...	4	0	seq
...........ugguuucacaugguggcuuaga..	55	0	seq
...........ugguuucacaugguggcuuagau...	31	0	seq
...........ugguuucacaugguggcuuagauu..	9	0	seq
...........ugguuucacaugguggcuuagauuu...	1	0	seq
...............uucacaugguggcuuaga..	1	0	seq
.................................uuGuuccaucuuuguau...............................	1	1	seq
.................................uuuuuccaucuuuguauU..............................	7	1	seq
.................................Cuuuuccaucuuuguauc..............................	1	1	seq
.................................uuuuuccaucuuuguauc..............................	34	0	seq
....uuuguaucuagcaccauuugaaaucaguguu.......	1	0	seq
....ucuagcaccauuugaaaucagu..........	6	0	seq
....cuagcaccauuugaaau..............	1	0	seq
....cuagcUccauuugaaauc.............	1	1	seq
....cuagcaccauuugaaauc.............	58	0	seq
....cuaCcaccauuugaaauc.............	1	1	seq
....cuagcaccauuugaaaucG............	5	1	seq
....cuagcaccauuugaaauca............	31	0	seq
....cuagcaccauuugaaaucGg...........	3	1	seq
....cuagcaccauuugaaaucag...........	31	0	seq
....cuagcaccauuugaaaucagu..........	2923	0	seq

oar-miR-29b

```
cuucuggaagcugguuucacaugguggcuuagauuuuuccaucuuuguaucuagcaccauuugaaaucaguguuuuaggag
..................................................cuagcaccauuugaaaucGgu..........    31      1      seq
..................................................cuagcaccauuugaaaucagug.........    1508    0      seq
..................................................cuagcaccauuugaaaucagugu........    794     0      seq
..................................................cuagcaccauuugaaaucaguguu.......    3       0      seq
...................................................uagcaccauuugaaauU.............    4       1      seq
...................................................uagcaccauuugaaauA.............    1       1      seq
...................................................uagcaccauuugaaauc.............    102     0      seq
...................................................uagcaccauuugaaaucG............    44      1      seq
...................................................uagcaccauuugaaauca............    34      0      seq
...................................................uagcaccauuugaaaucag...........    58      0      seq
...................................................uagcaccauuugaaaucGg...........    201     1      seq
...................................................uagcacAauuugaaaucag...........    1       1      seq
...................................................uagcaccauuugaaaucGgu..........    698     1      seq
...................................................uagcaccauuugaaaucagu..........    2033    0      seq
...................................................uagcaccauuugaaaucagug.........    547     0      seq
...................................................uagcaccauuugaaaucagugu........    432     0      seq
...................................................uagcaccauuugaaaucaguguu.......    2125    0      seq
...................................................uagcaccauuugaaaucaguguuC......    1       1      seq
...................................................uagcaccauuugaaaucaguguuu......    8       0      seq
....................................................agcaccauuugaaaucagu..........    1       0      seq
....................................................agcaccauuugaaaucaguguu.......    5       0      seq
.....................................................gcaAcauuugaaaucag...........    1       1      seq
.....................................................gcaccauuugaaaucaguguu.......    1       0      seq
........................................................ccauuugaaaucaguguG.......    1       1      seq
```
